# Clinical outcomes of liver transplantation in human immunodeficiency virus/hepatitis B virus coinfected patients in China

**DOI:** 10.1186/s12879-024-09284-2

**Published:** 2024-04-08

**Authors:** Jianxin Tang, Ruihui Weng, Taishi Fang, Kangjun Zhang, Xu Yan, Xin Jin, Linjie Xie, Dong Zhao

**Affiliations:** 1 Department of Liver Surgery & Organ Transplantation Center, Shenzhen Third People’s Hospital, The Second Affiliated Hospital of Southern University of Science and Technology, National Clinical Research Center for Infectious Disease, Longgang District, Bulan Road 29#, 518000 Shenzhen, China; 2 Department of Neurology, Shenzhen Third People’s Hospital, The Second Affiliated Hospital of Southern, University of Science and Technology, 518000 Shenzhen, China

**Keywords:** Human immunodeficiency virus, Hepatitis B virus, End-stage liver disease, Liver transplantation, China

## Abstract

**Background:**

Highly active antiretroviral therapy (HAART) has been able to improve the immune system function and survival of human immunodeficiency virus (HIV) patients. However, Patients coinfected with HIV and hepatitis B virus (HBV) are more likely to develop end-stage liver disease (ESLD) than those infected with HBV alone. Consequently, liver transplantation is often required for these patients. This study evaluates the outcomes of orthotopic liver transplantation (OLT) of HIV-HBV coinfected patients in China.

**Methods:**

We conducted a retrospective analysis on all HIV-HBV coinfected patients that underwent OLT from April 1, 2019 to December 31, 2021 and their outcomes were compared to all HBV monoinfected patients undergoing OLT during the same period. Patient outcomes were determined, including cumulative survival, viral load, CD4 T-cell count and postoperative complications.

**Results:**

The median follow-up of HIV recipients was 36 months after OLT (interquartile range 12–39 months). Almost all patients had stable CD4 T-cell count (> 200 copies/ul), undetectable HBV DNA levels, and undetectable HIV RNA load during follow-up. The 1-, 2-, and 3-year posttransplant survival rates were 85.7% for the HIV group (unchanged from 1 to 3 years) versus 82.2%, 81.2%, and 78.8% for the non-HIV group. Cumulative survival among HIV-HBV coinfected recipients was not significantly different from the HBV monoinfected recipients (log-rank test *P* = 0.692). The percentage of deaths attributed to infection was comparable between the HIV and non-HIV groups (14.3% vs. 9.32%, *P* = 0.665). Post OLT, there was no significant difference in acute rejection, cytomegalovirus infection, bacteremia, pulmonary infection, acute kidney injury, de novo tumor and vascular and biliary complications.

**Conclusions:**

Liver transplantation in patients with HIV-HBV coinfection yields excellent outcomes in terms of intermediate- or long-term survival rate and low incidence of postoperative complications in China. These findings suggest that OLT is safe and feasible for HIV-HBV coinfected patients with ESLD.

**Trial registration:**

Chinese Clinical Trial Registry (ChiCTR2300067631), registered 11 January 2023.

## Background

Highly active antiretroviral therapy (HAART), introduced in 1996, has significantly improved the survival of patients infected with the human immunodeficiency virus (HIV) [1]. It is well-established that HAART can suppress HIV replication, enhance immune function, and reduce opportunistic infections. With effective antiretroviral therapy, HIV infection has become a chronic disease, and the clinical comorbidities are increasing [2]. Among HIV-infected patients in China, HBV coinfection rates range between 9.5 and 14.5%, and the prevalence of (hepatitis B virus) HBV surface antigen (HBsAg) is estimated to be 13.7% [3, 4]. Liver-related mortality in HIV patients with viral hepatitis has become a major cause of death in many countries. Thus, end-stage liver disease (ESLD) has become the leading cause of death in HIV patients induced by HBV coinfection [5]. Besides, it is widely thought that HIV coinfection accelerates the course of liver disease and increases mortality. Despite recent advances in treating chronic hepatitis B, liver transplantation (LT) remains the last resort for patients with ESLD [6].

HIV infection has long been considered an absolute contraindication to liver transplantation due to this patient population’s relatively shorter life expectancy. Although the past decade has witnessed significant research progress, the clinical efficacy of LT has not been established. HIV infection and rejection-resistant immunosuppression after LT expose HIV recipients to serious complications, especially opportunistic infections. However, the advent of HAART has improved the prognosis for HIV-infected patients and encouraged many transplant centers to accept HIV-positive candidates. Several studies on outcomes of HIV-positive patients after LT have demonstrated stable HIV infection, survival, and complication rates comparable to HIV-negative patients [7–9].

To our knowledge, this is the first retrospective analysis of HIV-infected Chinese patients with HBV-related ESLD who underwent liver transplantation in the HAART era. Importantly, we evaluated the outcomes of all liver transplantations in HIV-positive patients and compared them with HIV-negative ones.

## Materials and methods

### Study design and participants

**Fig. 1:**
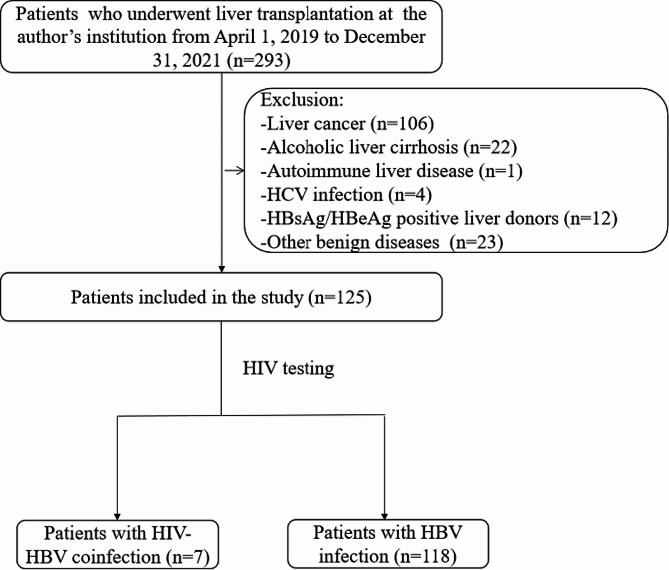
The design flow chart of the study

A retrospective, analytical and unicentric study was performed, and the design scheme is shown in Fig. 1. The study protocol followed the principles of the Declaration of Helsinki and was approved by the Clinical Research Ethics Committee of Shenzhen Third People’s Hospital (No. 2022-038-02). The study has been registered in Chinese Clinical Trial Registry (ChiCTR2300067631). All patients signed an informed consent form before liver transplantation.

We identified all patients (*n* = 293) who underwent a total orthotopic liver transplantation (OLT) for ESLD between April 1, 2019 to December 31, 2021. We excluded patients younger than 18 years, and those underwent combined transplantation (liver-kidney) or transplantation for primary liver cancer, alcoholic liver cirrhosis, HCV, autoimmune liver disease, other benign diseases or without known ESLD. Clinical data were collected, including sex, age, etiology of liver disease, HIV viral load, CD4 T cell counts, and model of end-stage liver disease (MELD) score. HIV patients that were placed on the waiting list for OLT had similar characteristics to non-HIV ones. In addition, the criteria for efficacy in HIV patients included a stable CD4 T-cell count and serum HIV RNA levels < 500 copies/mL. Our multidisciplinary team, including infectious disease specialists, hepatologists and transplant surgeons, was in charge of selecting patients on the waiting list.

### Liver transplantation and immunosuppressive therapy

Liver grafts were obtained from cadaveric donors. All donors were HIV and HBV/HCV-negative. The surgical technique of LT was a modified piggyback technique with triangulation of the hepatic veins. The liver-transplantation procedure and immunosuppressive regimen have been described in our previous published articles [10]. Besides, 2 patients with ABO-incompatible liver transplantation received additional induction therapy, including plasmapheresis twice, and rituximab 375 mg/m2 in two divided doses was administered intravenously before surgery. The immunosuppressive regimen used after liver transplantation was documented in all HIV patients.

### HAART Therapy

All patients receiving HAART had documented treatment before and after LT. Instead of using a standard antiretroviral regimen, each patient received an individualized treatment regimen pattern based on tolerability, genotypic sensitivity of the HIV, and physician preference. However, treating HBV infection in a coinfected patient with lamivudine or tenofovir alone can result in HIV resistance to these drugs, which may affect anti-HIV treatment options. All changes in HAART treatment after LT were recorded. Subsequently, some post-LT patients with HIV were switched to albuvirtide and dolutegravir, which have low hepatorenal toxicity and are not CYP3A4 enzyme inhibitors, reducing the impact of calcineurin inhibitor-type immunosuppressive drugs [11–12].

### Infection prophylaxis

Given the immunodeficiency from HIV and the antirejection medication, infection prophylaxis is critical. Preoperative prophylaxis was based on a regimen of third-generation cephalosporin plus lactamase inhibitor (3 g/day) from induction to POD 14. Caspofungin (50 mg/day), which has a low effect on tacrolimus concentrations, was administered as an antifungal prophylaxis on the first postoperative day until 2 weeks after surgery. Oral co-trimoxazole for the first 3 months to prevent Pneumocystis jirovecii prophylaxis. For cytomegalovirus prophylaxis, ganciclovir 5 mg/kg daily was given for 2 weeks after liver transplantation. When cytomegalovirus antigen level > 10 was positive, ganciclovir 10 mg/kg/day was administered intravenously (IV) until cytomegalovirus antigen negativity. The antibiotic treatment plan was changed according to the postoperative infection etiological detection and drug susceptibility test.

### Prophylaxis against hepatitis B virus recurrence

All patients received long-term passive immunization to prevent hepatitis B virus recurrence after LT. The regimen for prevention of HBV recurrence after LT was as follows: intraoperative administration of 4,000 IU of hepatitis B immune globulin (HBIG) intravenously during the anhepatic phase of liver transplantation, followed by 2,000 IU daily for the first 7 postoperative days. Subsequently, the titer of hepatitis B surface antibody (HBsAb) was maintained above 500 IU/ml within the first postoperative month. Immunoprophylaxis was continued subsequently and throughout follow-up, with monthly intramuscular injections of HBIG 400 to 600 IU to keep the titer of HBsAb greater than 100 IU/ml. From 2019, all patients who underwent LT due to HBV infection received dual immunization prophylaxis with HBIG and nucleoside (acid) analogs. Resumption of oral antiretroviral drugs occurred on day 2 after LT.

### Monitoring of graft liver

Regular graft liver biopsies were performed in all patients after intraoperative donor liver blood recirculation, 6 months after liver transplantation, and then annually after liver transplantation. If the result of the liver function examination was abnormal, an additional liver biopsy was required. Each liver biopsy tissue was fixed and paraffin-embedded for histological examination. Diagnosis of acute or chronic rejection was based on the Banff classification.

### Postoperative follow-up

Postoperative follow-up schedule: Patients were followed 3 times weekly for the first 2 weeks, then once a week for the 1st month, every 2 weeks for the 3rd month, monthly for the 6th month, every 2 months at the end of the 1st year, and every 3 months for the second and third years. The follow-up plan was adjusted according to the patient’s condition. Post-transplant data collection included liver and kidney function tests, blood cell analysis, blood coagulation function, CD4 T cell counts, HIV-RNA, HBV-DNA, HBsAb levels, immunosuppressive doses and all clinically relevant events such as rejection and infectious complications.

### Statistical analysis

All statistical analyses were performed using SPSS 24.0 statistical software. Categorical variables were displayed as frequency (%), continuous variables as mean ± SD or median (interquartile range) used for the descriptive statistics, as appropriate. Group comparisons for categorical variables were performed using the χ²-test and for metric variables using the Mann-Whitney U test. Survival curves were drawn using the Kaplan-Meier method and compared with the log-rank test. A two-sided p-value < 0.05 was statistically significant.

## Results

### General clinical data

The study included 125 patients with HBV cirrhosis decompensation or liver failure who underwent OLT between April 1, 2019 to December 31, 2021. Seven were HIV/HBV coinfected patients (all male) with a median age of 52 years (interquartile range 46–61 years), including 2 patients with ABO-incompatible liver transplantation. 118 patients without HIV infection that underwent OLT during the same period were also included, and the clinical data are summarized in Table 1. There was no statistically significant difference in charateristic variables (age, sex, indication, HBV DNA load, and MELD score) between the HIV and non-HIV groups.

**Table 1:** Characteristics of HIV-HBV coinfected and HBV monoinfected patients

	HIV-HBV Coinfection (*n* = 7)	HBV Monoinfection (*n* = 118)	P value
Age (year) (median (IQR))	52(46–61)	48.5 (35–55)	0.323
Male (n (%))	7(100%)	103 (87.29%)	0.315
Indication (n (%))			
Liver failure	6(85.7%)	73(61.86%)	0.204
Decompensated liver cirrhosis	1(14.3%)	45(38.14%)	
Detectable HBV DNA pre-OLT (n (%))	4(57.1%)	101(85.59%)	0.046
MELD score (median (IQR))	34(31–40)	31(18-39.25)	0.544
CD4 cell count pre-OLT (cells/ul) (median (IQR))	121(42–165)	256.5(146.25–476.5)	0.006
Acute rejection (n (%))	1(14.3%)	5 (4.24%)	0.227
Complications (n (%))			
Bacteremia	1(14.3%)	10 (8.47%)	0.598
CMV infection	5 (71.43%)	47(39.83%)	0.099
Pulmonary infection	2(28.57%)	34 (28.81%)	0.989
Acute kidney injury	1(14.3%)	7(5.93%)	0.380
De novo malignancies	0	2(1.69%)	0.728
Biliary complications	0	6(5.08%)	0.541
Artery complications	0	2	0.728
Hepatic venous complications	0	1	0.807
Portal vein thrombosis	0	2(1.69%)	0.728
Length of hospital stay post-OLT (median (IQR))	37(31–64)	26.5 (20.75-35)	0.092
Duration of follow-up after LT (month)	36(12–39)	23.5(12.9–33.4)	0.345

Abbreviations: HBV, hepatitis B virus; CMV, Cytomegalovirus; HIV, human immunodeficiency virus; MELD, model for end stage liver disease; OLT, orthotopic liver transplantation; IQR, Interquartile range

### Patient and graft survival

**Fig. 2:**
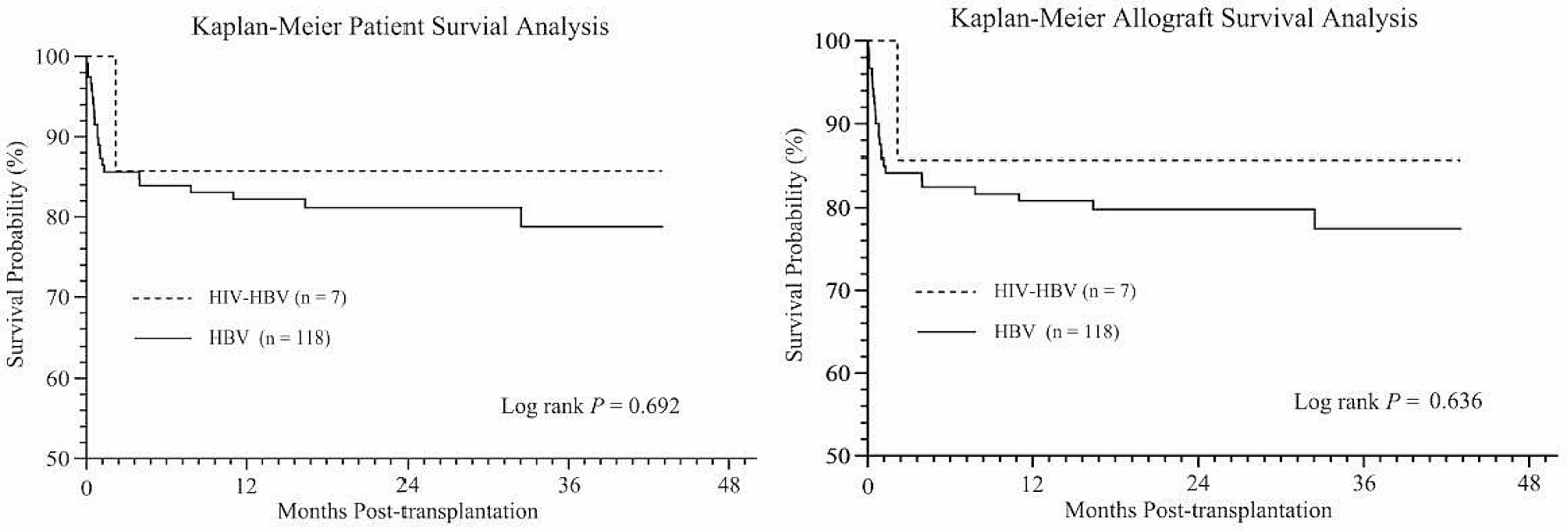
Kaplan-Meier curves illustrating (**A**) patient survival and (**B**) allograft survival in the liver transplant cohort, comparing HIV-HBV coinfected group with HBV monoinfected group. Log rank test *P* = 0.692 for patient survival and *P* = 0.636 for allograft survival. HBV, hepatitis B virus; HIV, human immunodeficiency virus

All HIV patients survived beyond 30 days following LT. The median follow-up of HIV recipients was 36 months after LT (interquartile range 12–39 months). As shown in Fig. 2A, the actual 1-, 2-, and 3-year survival rates were 85.7%, 85.7%, and 85.7% for the HIV group versus 82.2%, 81.2%, and 78.8% for the non-HIV group, respectively (log-rank test *P* > 0.05). The graft 1-, 2-, and 3-year survival rates were 85.7%, 85.7%, and 85.7% for the HIV group versus 80.8%, 79.8%, and 77.5% for the non-HIV group, respectively (*P* > 0.05, log-rank test) (Fig. 2B). The observed all-cause mortality was 14.3% (*n* = 1/7) in the HIV group compared to 19.5% in the non-HIV group (*n* = 23/118). One HIV recipient developed severe septicemia with progressive multiorgan failure and died 2 months after LT. In the non-HIV group, 23 deaths were due to septicemia (34.78%, *n* = 8), severe pneumonia (13.04%, *n* = 3), cardiovascular events (8.70%, *n* = 2), neurological events (13.04%, *n* = 3), gastrointestinal bleeding (13.04%, *n* = 3), de novo lung cancer (4.35%, *n* = 1), graft versus host disease (4.35%, *n* = 1), early allograft dysfunction (4.35%, *n* = 1) and disseminated intravascular coagulation (4.35%, *n* = 1). Of the 23 deaths in the non-HIV group, 17 (73.91%) occurred within 2 months after OLT.

### Rejection and immunosuppression

As shown in Tables 1 and 2 of 7 HIV-infected patients (14.29%) experienced acute allograft rejection. Acute rejection was observed in patient 1 at 2 weeks after liver transplantation. The Banff rejection activity index scores were 4 for patient 1, which suggested mild acute rejection. No patient had histological evidence of chronic rejection. A further course of methylprednisolone and an increased dose of tacrolimus and MMF to enhance baseline immunosuppression was required until graft liver function returned to normal. The only HIV-infected patient who died experienced severe pneumonia and infection post-LT. The infection in patient 7, with obvious bone marrow depression and leukopenia, was thought to result from excessive immunosuppression, and the tacrolimus dose was reduced or even stopped. This episode was unrelated to his demise, which occurred 2 months post-LT. The rejection rate was 14.29% in the HIV group and 4.24% in the non-HIV group, which was not statistically significant (*P* = 0.227).

**Table 2:** Data summary of patients with HIV-HBV coinfection before and after liver transplantation

Case	Age (year)	sex	Indication for OLT	HIV CDC classification Pre-OLT	CD4 cell count (cells/ul) Pre-OLT	HIV-RNA (copies/ml) Pre-OLT	HBV-DNA (copies/ml) Pre-OLT	MELD score	HAART Pre-OLT	Latest CD4 cell count (cells/ul)	Latest HIV-RNA (copies/ml)	Latest HBV-DNA (copies/ml)	HAART after OLT	Hospital stays (d)	Complications	Follow-up (mo)	Outcome
1	61	Male	HBV-ACLF	A3	42	ND	4.23E + 06	40	DTG + 3TC + TAF	209	ND	< 20	DTG + 3TC + TAF	37	Acute rejection	43	Alive
2	52	Male	HBV-DLC	C3	140	< 500	6.71E + 02	10	FTC + TAF + RAL	317	ND	< 20	FTC + TAF + RAL	18	No	39	Alive
3	47	Male	HBV-ACLF	A3	181	< 500	4.51E + 04	35	TAF + FTC + DTG	478	< 250	< 20	TAF + FTC + DTG	39	No	37	Alive
4	46	Male	HBV-ACLF	A3	121	< 500	< 200	40	DTG + ABT	476	ND	< 20	DTG + ABT + TAF	64	No	36	Alive
5	34	Male	HBV-ACLF	A3	113	< 500	< 200	31	3TC + DTG	244	< 250	< 20	B/F/TAF	31	No	15	Alive
6	65	Male	HBV-ACLF	A3	165	< 500	2.53E + 04	34	TDF + FTC + RAL	231	< 250	< 100	TDF + FTC + RAL	31	No	12	Alive
7	58	Male	HBV-ACLF	A3	38	< 500	< 100	31	3TC + DTG	3	< 500	< 100	DTG + ABT + TAF	66	Pulmonary infections, acute renal failure	2	Dead

Abbreviations: 3TC, lamivudine; ACLF, acute-on-chronic liver failure; ABT, Albuvirtide; B/F/TAF, Bictegravir/emtricitabine/tenofovir alafenamide; DLC, decompensated liver cirrhosis; DTG, dolutegravir; FTC: Emtricitabine; HIV, human immunodeficiency virus; HBV, hepatitis B virus; MELD, model for end stage liver disease; OLT, orthotopic liver transplantation; RAL, Raltegravir; TAF, tenofovir alafenamide; TDF, Tenofovir disoproxil fumarate; IQR, Interquartile range; ND, Not detectable;

All 7 HIV-infected patients received immunosuppression with steroids, tacrolimus, and mycophenolate mofetil. During the follow-up, patients 1, 2 and 3 were switched from tacrolimus to sirolimus due to impaired renal function and patient 6 due to neurologic toxicity. As described above, patient 1 received steroid pulses therapy (80 mg methylprednisolone for 3 days, then tapered) and 200 mg rituximab twice approximately 2 weeks after ABO-incompatible LT due to high anti-A IgG and IgM titers (1:64 and 1:16, respectively) and acute cellular rejection. Liver enzymes decreased and subsequently remained in the normal range.

### Patient outcomes following HAART

Of the 7 patients with HIV infection, 3 received more than 12 months of HAART prior to LT. Patients 6 and 7, receiving only 1 month of HAART, developed acute liver failure secondary to HBV and were unaware of HIV infection prior to their hospitalization. All patients receiving HAART had documented treatment before and after LT. Instead of using a standard antiretroviral regimen, each patient received an individualized treatment regimen pattern based on tolerability, genotypic sensitivity of the HIV, and physician preference.

All HIV-infected patients continued to receive HAART treatment on the second day after transplantation. Table 2 shows the antiretroviral and CD4 T-cell count and HIV load for each patient after OLT. Patient 1 had a low CD4 T-cell count (42 cells/ul) with an undetectable HIV load before OLT. However, with HAART, the HIV load was undetectable (< 50 copies/ ml), and the CD4 T-cell count was stable (> 200 cells/ ul) 43 months after OLT. Patients 2, 3 and 4 had stable CD4 T-cell count (> 200 cells/ul) and an undetectable HIV load (< 250 copies/ml) 3 years after OLT. The pretransplant antiretroviral therapy was continued until OLT. The HAART regimen was altered to completely suppress persistent low-level HIV replication, depending on the patient’s condition. Patient 1 was switched from FTC/TDF and DTG to 3TC, TAF and DTG 6 months before OLT. For antiretroviral therapy in patients with a low CD4 T-cell count and an undetectable HIV load, TAF combination therapy continued to be added after liver transplantation. Although HIV load could not be detected after liver transplantation, patient 5 had a low CD4 T-cell count, which remained above 200 cells/ul after switching to B/F/TAF. Patient 7 had an extremely low CD4 T cell count, with an undetectable HIV load after OLT. On the second day, the patient was switched to dolutegravir, albuvirtide, and tenofovir alafenamide. Patient 7 developed severe pneumonia 10 days after OLT. At the time of death, the patient had an HIV viral load of < 500 copies/ml and a CD4 T-cell count of 3 cells/ul. The patient exhibited a progressive course of progression on HAART and low-dose immunosuppressive therapy until he died of sepsis and multiple organ failure 66 days after OLT.

### Incidence of infection after OLT

CMV viremia was detected in 5 patients and treated with intravenous ganciclovir. There was no significant difference in the percentage of patients with CMV viremia infection between the HIV group (71.43%, 5 of 7) and the non-HIV group (39.83%, 47 of 118). One HIV recipient developed severe septicemia and severe pneumonia 10 days after OLT. Pathogenic culture and next-generation sequencing of alveolar lavage fluid and blood samples yielded carbapenem-resistant Pseudomonas aeruginosa and Aspergillus. According to drug sensitivity results, voriconazole, amphotericin B liposome, ceftazidime and amikacin were used for combined treatment. Unfortunately, the patient died of sepsis with progressive multiorgan failure 2 months after transplantation. The observed percentage of HIV-infected patients who died of infection was 14.3% (*n* = 1/7) versus only 9.32% (*n* = 11/118) in the non-HIV group. The mortality rate due to infectious causes was comparable between the two groups (*P* = 0.665).

### Recurrence of viral hepatitis

Four HIV-infected patients had detectable HBV viral loads at the time of transplant. All four patients that underwent OLT for HBV-related liver disease had undetectable HBV DNA by polymerase chain reaction after surgery. The HBV-DNA levels were less than 100 copies/ml in serum samples from all 7 patients after liver transplantation and remained undetectable during the follow-up period. HBsAg became undetectable within 2 days of liver transplantation. All patients were HBeAg negative after liver transplantation to the end of follow-up. Details of HBV prophylaxis with hepatitis B immunoglobulin in combination with nucleoside or nucleotide analogs are shown in Table 3.

**Table 3:** Recurrence and prevention of hepatitis B in HIV-positive patients after OLT

Case	Viral hepatitis	HBV therapy	HBV DNA detected	HBsAg	HBeAg
1	HBV	3TC/TAF, HBIG	negative	negative	negative
2	HBV	TAF, HBIG	negative	negative	negative
3	HBV	TAF, HBIG	negative	negative	negative
4	HBV	TAF, HBIG	negative	negative	negative
5	HBV	TAF, HBIG	negative	negative	negative
6	HBV	TDF, HBIG	negative	negative	negative
7	HBV	TAF, HBIG	negative	NA	NA

Abbreviations: 3TC, lamivudine; ETV: entecavir; HBeAg, hepatitis B e-antigen; HBIG, hepatitis B immune globulin; HBsAg: Hepatitis B surface antigen; TAF, tenofovir alafenamide; TDF, tenofovir disoproxil fumarate; NA, not available

## Discussion

In the HAART era, liver transplantation for HIV-infected patients is considered a reasonable choice. However, in the past, most transplant centers refused to accept HIV- infected patients for liver transplantation due to organ shortage and insufficient data. Liver transplantation has been performed in HIV-infected patients in several transplant centers in recent years [13]. Consistent with the literature, we substantiated that liver transplantation is feasible in this patient population; indeed, postoperative HAART therapy can suppress the viral load, stabilize CD4 T-cell count, and lead to no significant increase in opportunistic infections [14–16]. Few reports have involved liver transplantation outcomes in HIV/HBV coinfected patients. About 74 million people in China are carriers of the hepatitis B virus, which represents a serious public health issue [17]. Herein, we summarize the results of liver transplantation in HIV-positive patients with HBV from China.

The overall mortality rate for liver transplantation in HIV/HBV coinfected patients at our transplant center was 14.3% (1/7), lower than the mortality rate reported by a Spanish transplant center (38%) and in HIV-infected patients transplanted in Germany (41%) [18, 19]. Our study found 85.7% survival of both patient and graft after a mean follow-up of 36 months (with a maximum follow-up of 43 months), demonstrating the applicability of liver transplantation in this subgroup of patients. Compared with previous studies, the lower mortality rate in HIV/HBV coinfected patients is closely related to the development of efficient antiretroviral therapy, which has low drug resistance, high efficacy, and minimal interaction with commonly used immunosuppressive drugs. In our study, HIV patients were treated with INSTIs after OLT, especially second-generation INSTIs. In addition, the low mortality rate is also influenced by factors such as improvements in liver transplant surgery techniques, perioperative management, and antibiotic upgrades. HIV monoinfection or opportunistic infection does not appear to be a significant risk factor for patient survival after transplantation [20]. Current evidence suggests bacterial infection and sepsis are the leading causes of death after liver transplantation, especially in the early post-transplantation period [21, 22]. Patient 7, who had an HIV load < 50 copies/ml but a low CD4 T-cell count before LT, died of multiple organ failure from bacterial infection and sepsis 66 days after LT. In contrast, the CD4 T-cell count of patient 1 was less than 50 cells/ ul before LT, but the HIV disease was stable, and the patient remained alive after LT. He had an undetectable HIV load (< 50 copies/ ml) and a stable CD4 T-cell count (> 200 cells/ ul), with no opportunistic infection 43 months after LT. This indicates that a T-cell count of fewer than 100 cells/ ul is not an absolute contraindication of liver transplantation without definite infection. These discrepancies may reflect differences in postoperative infection prophylaxis and immunosuppressive management and underline the importance of aggressive infection-prevention therapy and avoidance of excessive immunosuppression early after transplantation in HIV/HBV coinfected patients.

HCV coinfection remains a key factor in the mortality of HIV-positive patients after liver transplantation in European and American HIV-positive patients [23, 24]. HCV recurrence in HIV patients is more aggressive, and liver fibrosis is more rapid due to HAART toxicity [25]. An increasing body of evidence suggests that treatment for HCV recurrence positively affects graft survival and mortality [26, 27]. Unlike HCV coinfection, HIV-infected patients with HBV appear to have better outcomes after OLT when HBV reinfection prophylaxis is properly provided [28]. Notably, there was no significant difference in survival between HIV/HBV coinfected patients and HBV monoinfected patients after liver transplantation. Anadol et al. showed that the 5-year survival rate of HIV/HBV-coinfected patients was 80%, and none of the HIV/HBV-coinfected patients developed clinically relevant HBV-related end-stage liver disease after liver transplantation [19]. The best option for preventing recurrent HBV infection in HIV/HBV coinfected patients appears to be combining pretransplantation and posttransplantation antiviral therapy with HBIG administration [29]. In our study, all patients received prophylaxis in combination with HBIG and antiviral therapy. Consistent with findings reported by Tateo et al. [30], all HBV/HIV coinfected patients were HBsAg-negative and HBV-DNA below 100 IU/ml. Interestingly, despite the successful prevention of recurrent hepatitis B, low levels of HBV-DNA were detected in approximately 50% of HBV/HIV coinfected patients treated with this combination regimen, and an 85% patient survival rate was achieved at 4 years of follow-up [31].

In the early posttransplant period, graft function and rejection prevention are major determinants of the outcomes of HBV/HIV coinfected patients instead of HIV infection. In addition, although HIV patients are considered immunosuppressed, there is an additional issue in managing these patients that may lead to higher rejection rates [32]. In the present study, 1 case of mild acute rejection was identified in HIV patients (14.3%) compared to 4.24% in non-HIV patients with no significant difference. All rejection episodes were easily treated according to routine protocols as previously described. Patient 7 was transferred to the intensive care unit due to severe pulmonary infection 10 days after the operation, and the concentration of tacrolimus and the dose of MMF were reduced accordingly. Two weeks after the operation, the liver function gradually deteriorated, and a liver biopsy showed acute cholestatic hepatitis with inflammation and necrosis equivalent to G3, moderate intrahepatic cholestasis, and no indication of acute rejection. Due to sepsis and multiple organ dysfunction, the patient was treated with low-dose methylprednisolone antirejection therapy. This episode was unrelated to his demise, which occurred 2 months post-LT. Coffin et al. showed that the acute rejection rates in HIV/HBV coinfected patients and monoinfected HBV patients were comparable (22.7% (*n* = 5/22) vs. 10% (*n* = 2/20), *p* > 0.05) [31].

Postoperative infection of HIV patients is also a key concern in this patient population. Although the prevalence of CMV viremia in patients with advanced HIV infection remains high, good immune recovery by antiretroviral treatment is sufficient to suppress CMV viral levels without increasing the risk of CMV end-organ disease [33]. Based on the medical literature, there has been no increase in the incidence rate of transplant or HIV-related opportunistic infections. The incidence of CMV and other opportunistic infections was not different between the HIV-positive and HIV-negative groups. More importantly, HIV did not appear to progress after liver transplantation in the post-HAART era. CD4 T-cell counts and HIV viral loads were stable in most patients as long as HAART could be administered [34]. In our study, there was no significant difference in the incidence of CMV viremia, bacteremia, and pulmonary infection between the HIV group and the non-HIV group. The comparable mortality rates attributed to infection between the HIV and non-HIV groups suggest that the risk of opportunistic infection after liver transplantation is not increased under HAART treatment.

The optimal management of the HAART regimen after OLT has not yet been determined. Pharmacological interactions between calcineurin inhibitors and HAART regimens containing protease inhibitors have been documented [35, 36]. Potential drug interactions must be considered when considering a specific antiretroviral regimen. Protease inhibitors (PI) are a part of most HAART regimens [37]. It is well known that PIs inhibit CYP3A, a component of the cytochrome P450, which results in markedly prolonged half-lives of the calcineurin inhibitors and sirolimus [38]. Accordingly, we must consider the optimal timing of HAART initiation, drug interactions between HAART and immunosuppressive regimen, and the control of disease recurrence after transplantation. All 7 Chinese patients with HIV/HBV coinfection began to receive HAART treatment early, within 2 days after OLT. The choice of drugs for immunosuppression and antiretroviral therapy is another key factor. Potential hepatotoxicity must be considered in the selection of HAART regimens to reduce liver-related mortality, such as stavudine (D4T) [39], azidothymidine (AZT) [40] or didanosine (ddI) [41]. All Chinese patients did not receive the above HAART drugs. In addition, HAART drugs with minimal interactions with other drugs must be considered. Subsequently, some post-LT patients with HIV were switched to albuvirtide and dolutegravir, which have low hepatorenal toxicity and are not CYP450 enzyme inhibitors, reducing the impact of calcineurin inhibitor-type immunosuppressive drugs. In this study, the HIV RNA load could not be detected during follow-up after LT. However, the adverse effects of HAART combined with immunosuppressive drugs on graft survival, the right time and dose of HAART after LT and the frequency of other secondary complications need to be further evaluated.

In conclusion, our data suggest that liver transplantation for patients with HIV/HBV coinfection represents the only way to survive from decompensated cirrhosis or liver failure. The outcome of these recipients is highly dependent on the patient’s state at the time of transplantation. Patient 1 in this study had a CD4 T-cell count of less than 100 cells/ul, which is not an absolute contraindication of liver transplantation without an established infection. The acceptable survival rate and control of HIV/HBV replication corroborate that the strategy of providing liver transplantation for HIV/HBV-coinfected patients with acute liver failure and end-stage liver disease is reasonable in China. Additional studies must be performed to determine medium- or long-term survival and improve post-transplant management to balance complex interaction factors, such as HAART and immunosuppressive drug selection, optimal treatment timing and dose adjustment.

## Data Availability

The datasets generated and/or analyzed during the current study are not publicly available as they contain information that could compromise participant privacy and consent but are available from the corresponding author on reasonable request.

## Abbreviations

3TC: Lamivudine
ACLF: Acute-on-chronic liver failure
B/F/TAF: Bictegravir/emtricitabine/tenofovir alafenamide
CMV: Cytomegalovirus
DLC: Decompensated liver cirrhosis
ESBL: End-stage liver disease
FTC: Emtricitabine
HAART: Highly active antiretroviral therapy
HBV: Hepatitis B virus
HBeAg: Hepatitis B e-antigen
HBIG: Hepatitis B immune globulin
HBsAg: Hepatitis B surface antigen
HIV: Human immunodeficiency virus
MELD: Model for end stage liver disease
MMF: Mycophenolate mofetil
OLT: Orthotopic liver transplantation
POD: Postoperative day
TAF: Tenofovir alafenamide
TDF: Tenofovir disoproxil fumarate
